# Non-traumatic Myositis Ossificans Mimicking Infected Hip in Children: Two Case Reports and Review of the Literature

**DOI:** 10.7759/cureus.69990

**Published:** 2024-09-23

**Authors:** Mohammad Alananzh, Mohammad Abu Hilal, Samir Sakka, Osama Aldahamsheh, Mohammad Alkhreisat

**Affiliations:** 1 Department of Special Surgery, Faculty of Medicine, Al-Balqa Applied University, Al Salt, JOR; 2 Orthopedic Surgery, Mutah University, Al Karak, JOR; 3 Department of Orthopaedics, Ibn Al-Haytham Hospital, Amman, JOR

**Keywords:** hip infection differential, hip joint swelling, myositis ossificans, nontraumatic ossification, pediatric hip pain

## Abstract

We present two pediatric cases of nontraumatic myositis ossificans (MO), which initially mimicked infections, leading to unnecessary treatments. The first case involves an 11-year-old boy with acute left hip pain and swelling, misdiagnosed as a hip joint infection and treated with antibiotics before histology confirmed MO. The second case is a two-year-old girl who presented with limping and restricted hip movement, initially suspected to have septic arthritis. Following MRI and clinical reassessment, her condition was diagnosed as MO. Both cases highlight the challenges of diagnosing MO in children and underscore the importance of including it in differential diagnoses for suspected infections.

## Introduction

Myositis ossificans (MO) is a benign reactive pseudotumor characterized by the formation of bone and cartilage matrix within skeletal muscles, most commonly near the hip and elbow joints. Despite its name, which implies an inflammatory or infectious origin, the pathogenesis of MO remains poorly understood. It is thought to result from the abnormal differentiation of fibroblasts into osteoblasts, often triggered by trauma or repetitive muscle overuse [1]. MO can be classified based on etiology, with Noble's classification [2] being widely recognized. This classification includes three types: myositis (fibrous) ossificans progressiva, traumatic MO circumscripta, and MO circumscripta without a history of trauma.

While nontraumatic MO is well-documented in adults [3,4], it is rare in children, with reported cases involving the neck, axilla, shoulder, upper limb, trunk, hip, thigh, and leg [5]. The rarity and variable clinical presentation of MO in pediatric patients often leads to diagnostic challenges, frequently resulting in misdiagnosis as infection and subsequent unnecessary treatments. Such mismanagement can heighten anxiety for both patients and their families. This paper presents two pediatric cases of nontraumatic myositis ossificans that initially mimicked infections, a phenomenon seldom documented in the literature [5]. These cases highlight the importance of considering MO in differential diagnoses to prevent unnecessary interventions.

## Case presentation

Case 1

**Figure 1:**
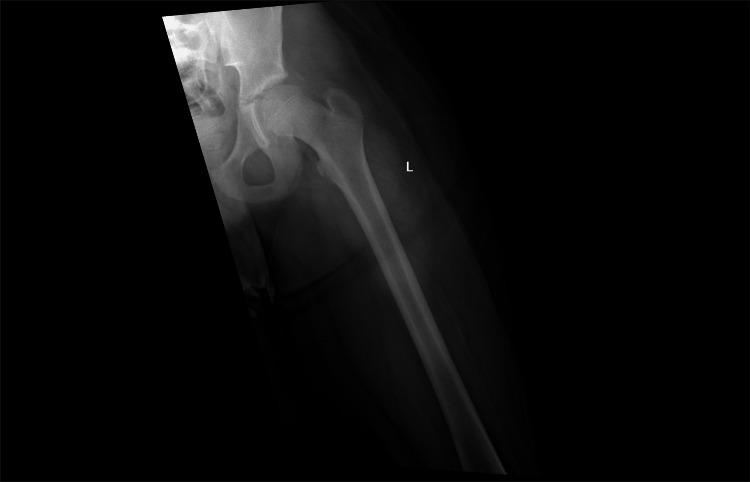
AP X-ray of the left hip

An 11-year-old boy presented to the emergency department with acute left hip pain and swelling. He has no previous or concurrent history of infection or trauma; however, he plays football occasionally at school. His initial examination showed anterolateral hip pain with limited hip range of motion, and his initial AP X-ray of the hip was unremarkable (Figure 1).

His blood tests are shown in Table 1.

**Table 1:** Blood test reslts for case one ESR: erythrocyte sedimentation rate; CRP: C-reactive protein

Test name	Result	Reference value	Unit
White cell count	10	4.5–13.5	10^9^/L
Neutrophils	70	35–80	%
ESR	20	≤20	mm/hr
CRP	35	≤6	mg/L

An initial diagnosis of a suspected infected hip joint led to the initiation of IV antibiotics. An initial MRI showed a periarticular abscess of the hip (Videos 1-2).

**Video 1:** Pelvis MRI STIR axial STIR: short tau inversion recovery

**Video 2:** Pelvis MRI STIR coronal STIR: short tau inversion recovery

**Figure 2:**
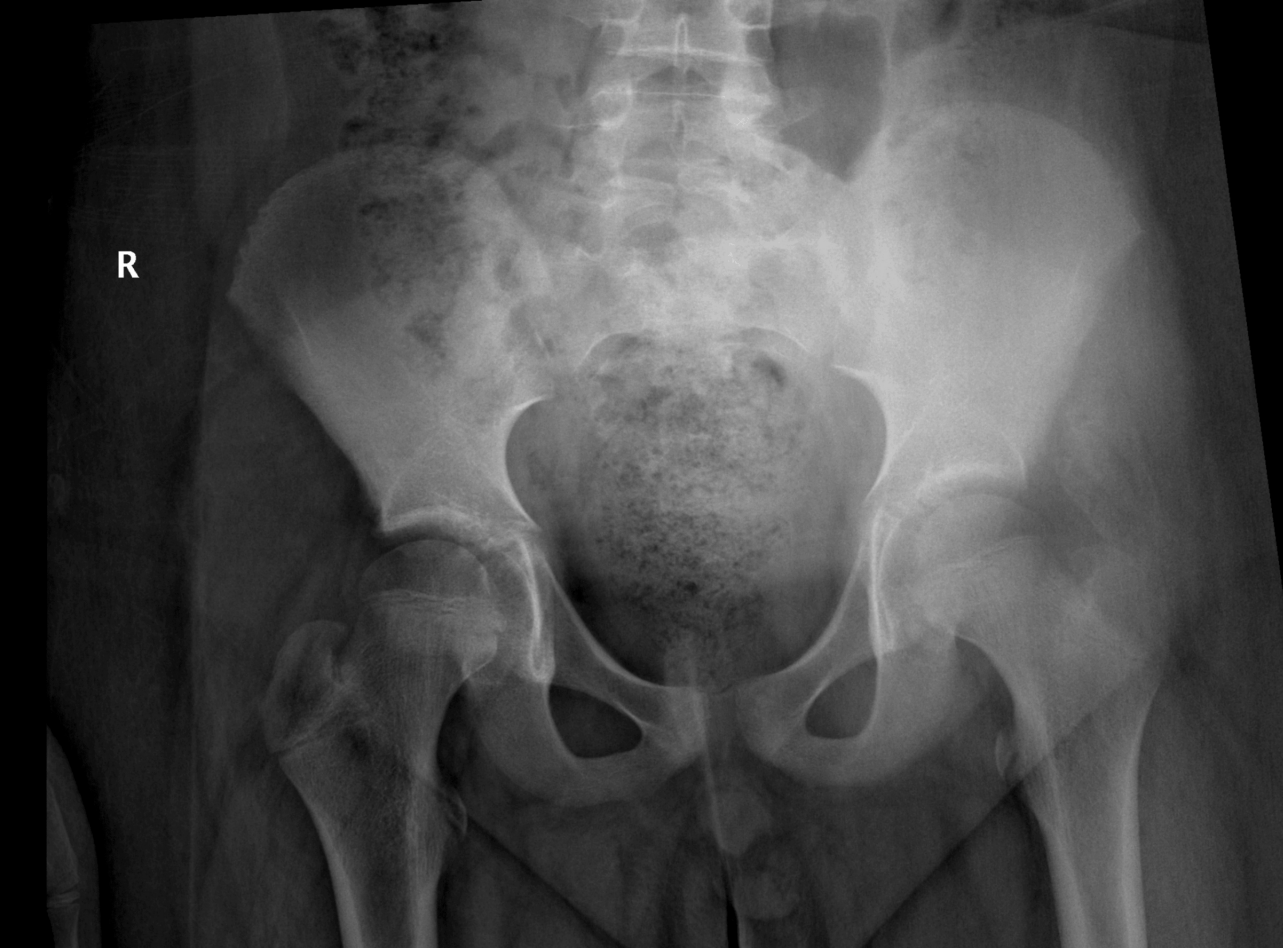
AP pelvis X-ray three months following presentation

The patient underwent surgical drainage, where a soft, inflamed muscle mass was found, and a tissue biopsy was taken. Then, the patient was continued on antibiotics and discharged with oral antibiotics and analgesia. The histology showed myositis ossificans. The child continued to have hip pain and stiffness. After six weeks, we sought a second opinion from a senior surgeon. Upon reviewing previous investigations, treatment, and a comprehensive examination, we observed pain within a moderate range of movement, along with pain-induced stiffness and localized tenderness on the lateral aspect of the hip. After a negative isotope scan, inflammatory markers, and muscle enzyme studies to exclude systematic muscle problems, they were reassured of the diagnosis as localized myositis ossificans affecting isolated muscle - Gluteus medius. An updated X-ray three months later showed the increasing calcification of the muscle (Figure 2).

**Figure 3:**
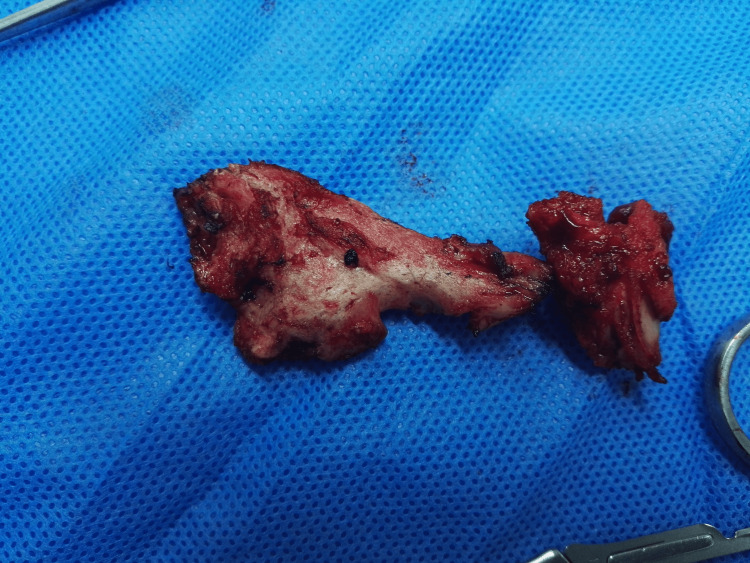
Image of surgical specimen following excision

He was treated with non-steroidal analgesia and local anesthetics with steroidal for pain until the maturation of the calcification at six months. After 18 months, when it became fully matured and well-defined, the bony mass was excised to reduce stiffness and pain (Figure 3).

Histopathology confirmed myositis ossificans. At the latest follow-up one year following surgery, the patient had no recurrence or further problems.

Case 2

A two-year-old female was admitted with a chief complaint of limping and decreased left hip movement. Three weeks prior to this admission, she was treated at another hospital for pneumonia with intravenous antibiotics and improved enough to be discharged after one week. Shortly thereafter, she developed an abscess at the cannula site on her right hand, which was managed with incision and drainage and regular wound care. One week following this, the patient began limping and exhibited restricted movement of the left hip. Upon presentation to our facility, she was afebrile, and a physical examination revealed an inability to bear weight and a left hip in a flexed position with restricted movement. Laboratory tests are shown (Table 2).

**Table 2:** Blood test reslts for case two ESR: erythrocyte sedimentation rate; CRP: C-reactive protein

Test name	Result	Reference value	Unit
White cell count	7.17	5–14.5	10^9^/L
Neutrophils	74	35–80	%
ESR	83	≤20	mm/hr
CRP	3.43	≤6	mg/L

**Figure 4:**
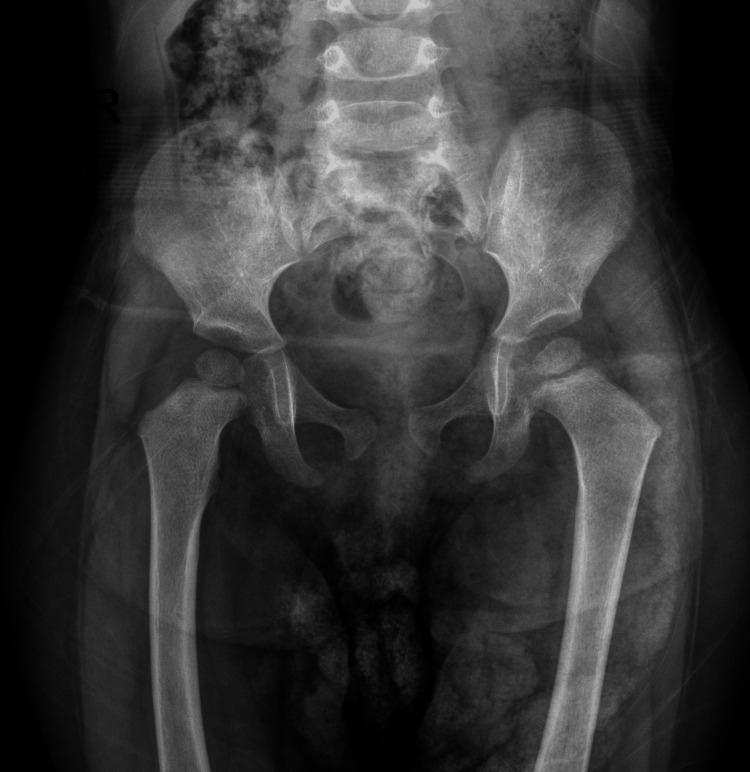
AP pelvis X-ray at presentation

The initial X-ray of the hip was unremarkable (Figure 4).

Given the clinical presentation and history, septic hip arthritis was suspected. The patient was admitted, and intravenous antibiotics were initiated. An MRI of the left hip was performed on the second day of admission, which revealed findings suggestive of myositis ossificans in the abductor muscles (Videos 3-5).

**Video 3:** Pelvis MRI coronal STIR STIR: short tau inversion recovery

**Video 4:** Pelvis MRI T1 axial

**Video 5:** Pelvis MRI STIR axial STIR: short tau inversion recovery

Given the absence of septic arthritis, the antibiotics were stopped based on clinical judgment, and the patient was managed with pain control and physical therapy to maintain joint function and mobility. At three weeks, the patient's symptoms completely resolved.

## Discussion

This report discusses two pediatric cases of hip pain, initially suspected to be caused by infection but ultimately diagnosed as MO, with one case requiring surgical excision. These cases underscore the diagnostic challenges MO presents in children and emphasize the importance of considering it in the differential diagnosis of suspected soft tissue or joint infections, particularly in the hip region.

A review of the literature on non-traumatic MO in children identified six cases where MO initially mimicked infection. These cases shared common features in terms of patient demographics, lesion locations, diagnostic approaches, treatments, and follow-up strategies. The patients ranged in age from 0 to 13 years, with lesions located in various regions, including the thigh [6,7], pectoralis [8], axilla [9], elbow [10], and leg [11]. Diagnostic imaging typically involves a combination of X-rays, ultrasound (US), CT, and MRI [5]. In most cases, treatment proceeded without a biopsy, although one case required an incisional biopsy [7] and another employed a percutaneous biopsy [10]. Surgical excision was the predominant treatment, though two cases were managed conservatively [6,11]. Follow-up was primarily clinical, focusing on recovery and monitoring for recurrence or complications (Table 3).

**Table 3:** Summary of the literature review XR: X-ray; US: ultrasound; CT: computed tomography, MRI: magnetic resonance imaging

	Author	Year	Sex	Age	Location	Imagery	Biopsy	Treatment	Follow-up
1	Jujena et al. [6]	2011	M	6 years	Bilateral thigh	XR, CT	None	Conservative	XR
2	Xia et al. [7]	2022	F	8 years	Thigh	XR, US, CT	Incisional	Excision	Clinical
3	Akatli et al. [8]	2021	F	13 years	Pectoralis	US, MRI	None	Excision	Clinical
4	Rehman et al. [9]	2021	F	12 years	Axilla	US, MRI	None	Excision	Clinical
5	Li et al. [10]	2016	M	9 years	Elbow	XR, CT	Percutaneous	Excision	Clinical
6	Krivec et al. [11]	2022	M	9 weeks	Leg	U/S XR MRI	None	Conservative	Clinical

The diagnosis of MO can be particularly challenging due to its evolving biochemical and imaging characteristics. Initially, alkaline phosphatase levels may be normal but gradually increase over a few weeks, peaking around week 10, and then normalizing by week 18 [12]. Calcium levels may initially decrease and then normalize before alkaline phosphatase levels begin to rise [12]. It is important to note that these findings are not observed in all cases. Inflammatory markers, such as CRP and ESR, also tend to increase in the early stages [13], which can further complicate the diagnosis in pediatric patients, as these symptoms can mimic infection.

Radiography may initially show no abnormalities or only soft tissue swelling [14]. A few weeks after the onset, imaging typically reveals a peripheral pattern of calcification with a low-density center, detectable by both CT and MRI [13,14]. In advanced stages, MRI often shows a well-defined heterogeneous mass with signal intensity resembling bone marrow and cortical calcification [14]. Due to the complexity of the presentation, additional diagnostic methods such as biopsy may be required to confirm the diagnosis, especially in the absence of a clear history of trauma.

Most cases of MO are self-limiting, so initial management is usually nonsurgical, focusing on pain control, preserving function, and alleviating symptoms [1,14]. Treatment typically involves a brief period of immobilization, followed by gentle range-of-motion exercises and adequate pain management, often with non-steroidal anti-inflammatory drugs (NSAIDs) [14]. Surgical intervention is generally reserved for cases that do not resolve on their own, particularly those with intractable pain or significant functional impairment, especially when MO is near a joint [1,14]. However, most treatment guidelines are based on studies involving adults and traumatic cases. Due to the rarity of MO in the pediatric population, further research is needed to determine whether these findings are applicable to children.

Early identification of MO is crucial to prevent unnecessary procedures and treatments, thereby reducing stress for both patients and their families. Based on the literature review and the observations from the two cases discussed, MRI has proven to be a valuable tool for diagnosing MO. However, when MRI and biochemical markers do not provide a definitive diagnosis, a biopsy may be necessary to confirm the condition. This approach ensures accurate diagnosis and appropriate management, which is particularly important given the challenges of diagnosing MO in children, where it can mimic other conditions such as infections or malignancies.

In our experience, one patient underwent surgical exploration to drain a suspected abscess, during which a mass was biopsied, leading to the diagnosis of MO. In the second case, having learned from the first, MO was considered in the differential diagnosis of an infected hip, thus avoiding unnecessary surgical intervention. Follow-up was primarily clinical, focusing on recovery and monitoring for recurrence or complications.

## Conclusions

The cases presented highlight the diagnostic complexity of MO in pediatric patients, where early symptoms can closely mimic common infectious conditions like septic arthritis. Misdiagnosis, as seen in these cases, can lead to unnecessary treatments, including antibiotics and surgical interventions, which are not only ineffective but also increase the risk of complications.

The differential diagnosis for a child presenting with hip pain includes septic arthritis, osteomyelitis, inflammatory conditions, hematoma, trauma, and myositis ossificans. Early MRI can guide the clinician to consider MO as part of the differential diagnosis. However, even with MRI, the findings may not always provide definitive diagnostic clarity, making close clinical monitoring essential in such cases.
